# The association of the advanced lung cancer inflammation index with the risk of *Helicobacter pylori* infection and mortality: a study of populations from two countries

**DOI:** 10.3389/fcimb.2026.1881537

**Published:** 2026-09-10

**Authors:** Qibo Hu, Huangxin Zhu, Qiao Wei, Qi Shen, Ming Xiong, Shen’an Huang

**Affiliations:** 1 Department of Gastroenterology, The Second Affiliated Hospital of Nanchang University, Nanchang, Jiangxi, China; 2 Department of Gastroenterology, Jiangxi Provincial Key Laboratory of Digestive Diseases, Jiangxi Clinical Research Center for Gastroenterology, Digestive Disease Hospital, The First Affiliated Hospital, Jiangxi Medical College, Nanchang University, Nanchang, Jiangxi, China; 3 Department of Intensive Care Unit, The First Affiliated Hospital of Xiamen University, Xiamen, Fujian, China

**Keywords:** advanced lung cancer inflammation index (ALI), *Helicobacter pylori (H. pylori)*, inflammation, nutritional status, survival analysis

## Abstract

**Background:**

*Helicobacter pylori* (*H. pylori*) infection is strongly linked to multiple diseases. Inflammation and nutritional status are intricately intertwined with *H. pylori* infection; however, most prior studies failed to integrate these two dimensions in a unified assessment.

**Methods:**

This study enrolled 3,718 eligible participants from the 1999–2000 National Health and Nutrition Examination Survey (NHANES). Multivariate logistic regression was utilized to examine the association between the ALI and *H. pylori* infection. Cox proportional hazards regression was employed to evaluate the association between ALI and all-cause mortality in *H. pylori*-infected patients. Restricted cubic splines (RCS) were implemented to characterize potential nonlinear relationships. Subgroup analysis explores the consistency of the associations among different populations. An independent duplicate cohort from the Second Affiliated Hospital of Nanchang University was additionally incorporated.

**Results:**

Elevated ALI emerged as a relevant factor for *H. pylori* infection. Higher ALI levels were associated with improved survival prognosis. Beyond an inflection point (log2ALI=5.44) this negative association gradually diminished. Subgroup analyses revealed that the interaction test showed that no significant interaction was found for all stratification factors. The association between ALI and *H. pylori* infection was consistent among the above subgroups. Independent duplicate cohort corroborated that as the level of log_2_ALI increases, the likelihood of *H. pylori* infection showed a monotonous increasing trend.

**Conclusion:**

Higher ALI levels were positively associated with an increased risk of *H. pylori* infection, and this association was consistent in an independent duplicate cohort. Among *H. pylori*-infected individuals, higher ALI was related to reduced all-cause mortality, with the association most prominent at lower ALI ranges.

## 1 Introduction

*Helicobacter pylori* (*H. pylori*) is a bacillus rich in urease, which decomposes urea to produce ammonia that neutralizes gastric acid, enabling it to adapt to the strong acidic environment of the stomach. Data from epidemiological investigations show that the worldwide rate of *H. pylori* infection stood at 43.1% across the past ten years, with a concurrent rate of 42.8% in China—both lower than the 51.3% recorded in the previous decade (Li et al., 2023; Xie et al., 2024). Even though the infection rate of *H. pylori* has gradually decreased in recent years, its harm has not diminished. *H. pylori* infection is not only linked to gastric illnesses including chronic gastritis, peptic ulcers and gastric cancer (Martin and Solnick, 2014), but also connected to many diseases outside the stomach, such as neurological, metabolic, allergic and cardiovascular disorders (Franceschi et al., 2014b; Santos et al., 2020; Sun et al., 2023). Given its close association with multiple tumors, *H. pylori* was classified as a Group I carcinogen by the World Health Organization as far back as 1994 (Vogiatzi et al., 2007).

Inflammation and nutritional conditions are closely associated with *H. pylori* infection. *H. pylori* strains that carry cytotoxin associated gene A (Cag A) can trigger intense local inflammatory responses and are closely linked to the onset of gastric cancer (Takahashi-Kanemitsu et al., 2020). Studies have shown that Cag A-positive *H. pylori* is strongly associated with biliary tract inflammation, thereby playing a critical role in cholangiocarcinoma progression (Boonyanugomol et al., 2011). *H. pylori*-associated gastritis causes abnormalities in common hematological inflammatory markers, such as neutrophils (NE), lymphocytes (LYC), and monocytes (Sağlam and Civan, 2023; Teng et al., 2023). Research indicates that numerous inflammatory indicators, including the Neutrophil to lymphocyte ratio (NLR) and platelet-to-lymphocyte ratio (PLR), may be related to *H. pylori* infection (Xiong et al., 2023). Other novel inflammatory markers, such as the neutrophil-albumin ratio (NAR) and systemic inflammation response index (SIRI), have also been confirmed to have a close connection with *H. pylori* infection (Qiu et al., 2025). These inflammatory indicators are also regarded as linked to the prognosis and all-cause mortality of individuals with *H. pylori* infection (Xiong et al., 2023; Qiu et al., 2025). Nutritional condition, evaluated through body mass index (BMI) and albumin (ALB), serves as another crucial factor in determining the severity of the disease (Franceschi et al., 2014a; Aimasso et al., 2019). A cross-sectional study demonstrated that abnormal BMI is associated with the occurrence of *H. pylori* infection (Siddiqui et al., 2018). Following *H. pylori* infection, changes in nutritional parameters like albumin may occur; after eradication of *H. pylori*, albumin levels significantly increase (Furuta et al., 2002).

However, earlier research only concentrated on either nutrition or inflammation, without integrating both for evaluation. The advanced lung cancer inflammation index (ALI), which is computed using BMI, ALB and NLR, reflects the interaction between nutritional status and inflammatory response, and initially played a significant part in predicting non-small cell lung cancer (Jafri et al., 2013). Later research has revealed that the ALI also holds significant value in evaluating the prognosis of various inflammation-associated disorders, including type 2 diabetes mellitus (Chen et al., 2023), arthritis (Xiu et al., 2025), chronic obstructive pulmonary disease (Xu et al., 2024), and fatty liver disease (Yu et al., 2025). Currently, the prognostic evaluation of ALI in *H. pylori* infection, gastritis, and related conditions remains to be further investigated.

Furthermore, we also attempted to analyze the factors, such as blood glucose level, which may be linked to ALI and *H. pylori* infection and its prognosis. The HbA1c, which reflects the average blood glucose concentration over the previous two to three months, is widely applied in clinical settings to evaluate glycemic control in diabetic patients (Lin, 2024). As a composite indicator, ALI integrates inflammatory response and nutritional status. Chronic inflammatory states and malnutrition can lead to insulin resistance, thereby increasing HbA1c levels (Szukiewicz, 2023; Zhu et al., 2025). Additionally, hyperglycemia can impair immune function, exacerbate gastric mucosal injury, and affect prognosis (Lee et al., 2024; Yin et al., 2025). Therefore, HbA1c may be a biologically reasonable intermediary factor that can link ALI with the risk and prognosis of *H. pylori* infection.

This study used data from the National Health and Nutrition Examination Survey (NHANES) to achieve the following objectives: (1) Investigate the association between ALI and the prevalence and mortality of *H. pylori* infection; (2) Explore the potential path of HbA1c in the associations between ALI and *H. pylori* positivity, as well as all-cause mortality. Additionally, data from the Second Affiliated Hospital of Nanchang University will be used as independent duplicate cohort to analyze the association of ALI and *H. pylori* infection.

## 2 Methods

### 2.1 Study population

**Figure 1:**
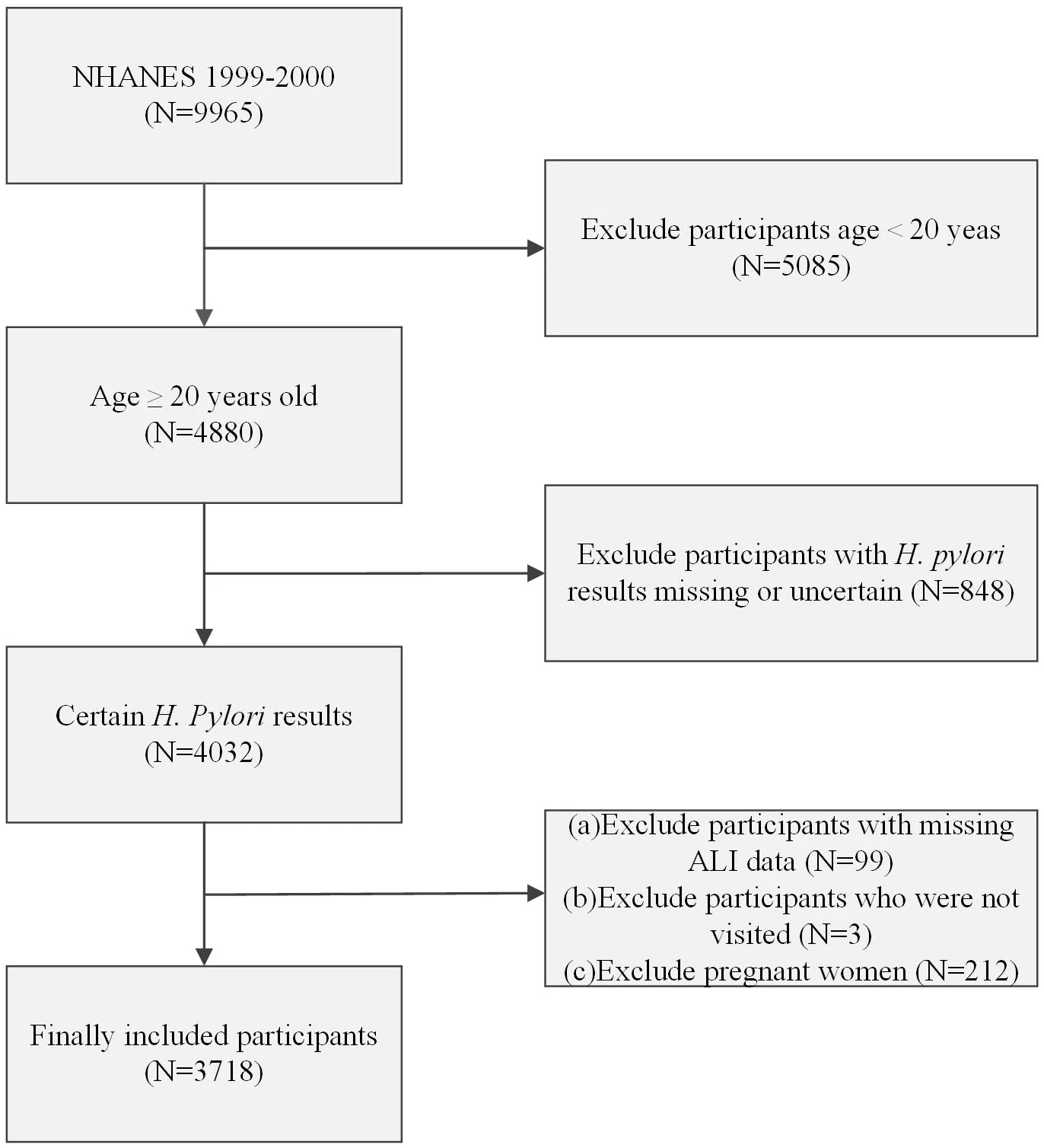
The flow chart of the study.

This study analyzed 9,965 participants from the 1999–2000 cycle of the NHANES database, as this was the only cycle containing *H. pylori* IgG results. We followed the detailed inclusion and exclusion pathway illustrated in **Figure 1**. After excluding a total of 6,247 participants (including 5,085 aged < 20 years, 848 with missing or indeterminate *H. pylori* results, 99 with incomplete ALI data, 3 lost to follow-up, and 212 pregnant women), a final total of 3,718 qualified participants were retained for analysis. For conducting consistency verification, we concurrently enrolled patients hospitalized in the Department of Gastroenterology of the Second Affiliated Hospital of Nanchang University between June 1, 2025, and November 30, 2025, who underwent *H. pylori* testing. We excluded individuals younger than 20 years old, those with incomplete data, and patients with a history of cirrhosis or malignant tumors, leaving 575 patients.

### 2.2 Assessment of *H. pylori* infection

Within the NHANES database, *H. pylori* infection status was determined based on antibody titers. According to previously established criteria (Meier et al., 2020), a titer < 0.90 was defined as negative, > 1.10 as positive, and values within the range of 0.9–1.1 were considered indeterminate. In the independent duplicate cohort, *H. pylori* infection was defined as a positive outcome on either the Carbon-13 urea breath test (13C) or Carbon-14 urea breath test (14C) (de Brito et al., 2019). Specifically, breath samples were collected from fasting individuals. For the 13C test, patients were administered a C13 capsule orally and waited for 30 minutes, after which a second breath sample was collected to complete the test. Alternatively, for the 14C test, patients ingested a C14-urea capsule with warm water and rested for 15 minutes. They then blew steadily into a collection tube for 1 to 3 minutes, after which the gas card was placed into a detector to finish the test.

### 2.3 Assessment of ALI

The calculation of ALI incorporates BMI, ALB, neutrophil count, and lymphocyte count. The ALI was calculated using the formula: ALI = BMI (kg/m^2^) × ALB(g/dL)/NLR, where NLR = Neutrophil count (10^9^/L)/Lymphocyte count (10^9^/L).

### 2.4 Assessment of mortality

This research assessed mortality outcomes among individuals with *H. pylori* infection, with a primary focus on all-cause mortality. Survival information was obtained by matching NHANES participant records with the National Death Index; the observation period ran from study enrollment up to December 31, 2019, or until the time of death. Follow-up duration was calculated in months to support precise survival analysis.

### 2.5 Potential covariates

This study incorporated sociodemographic characteristics, lifestyle factors, self-reported medical histories, and laboratory measurements. Sociodemographic variables included age, gender, race/ethnicity (Mexican American, non-Hispanic White, non-Hispanic Black, or other), education level (high school or lower, college or higher), marital status (married or living with a partner, widowed/divorced/separated, or never married), and economic situation reflected by the poverty-income ratio (PIR). Age was categorized into ≥ 60 years (elderly group) and < 60 years (younger and middle-aged group). Economic status was divided into impoverished (PIR < 1) and non-impoverished (PIR ≥ 1) (Huang et al., 2026; Zhu et al., 2026a). Lifestyle factors included smoking status and alcohol consumption status. Smoking status was classified into three groups: never smokers, former smokers, and current smokers. Alcohol consumption was defined as consuming > 12 drinks per year. Self-reported medical history included hypertension and diabetes. Hypertension was defined by a physician’s diagnosis, current use of antihypertensive drugs, or repeated measurements showing systolic blood pressure ≥140 mmHg or diastolic blood pressure ≥ 90 mmHg (Jones et al., 2025). Diabetes was defined as physician-diagnosed disease, current use of hypoglycemic agents, fasting plasma glucose ≥ 126 mg/dL (7.0 mmol/L), or HbA1c ≥ 6.5% (Xiao et al., 2025). Laboratory tests included serum creatinine (SCR, mmol/L), total cholesterol (TC, mmol/L), high-density lipoprotein (HDL, mmol/L), serum uric acid (SUA, mmol/L), and C-reactive protein (CRP, mg/L).

### 2.6 Collection of clinical indicators

During patient information collection, data on age, gender, education level, marital status, height, weight, smoking and drinking status, as well as hypertension and diabetes history were gathered.

### 2.7 Statistical analysis

**Figure 2:**
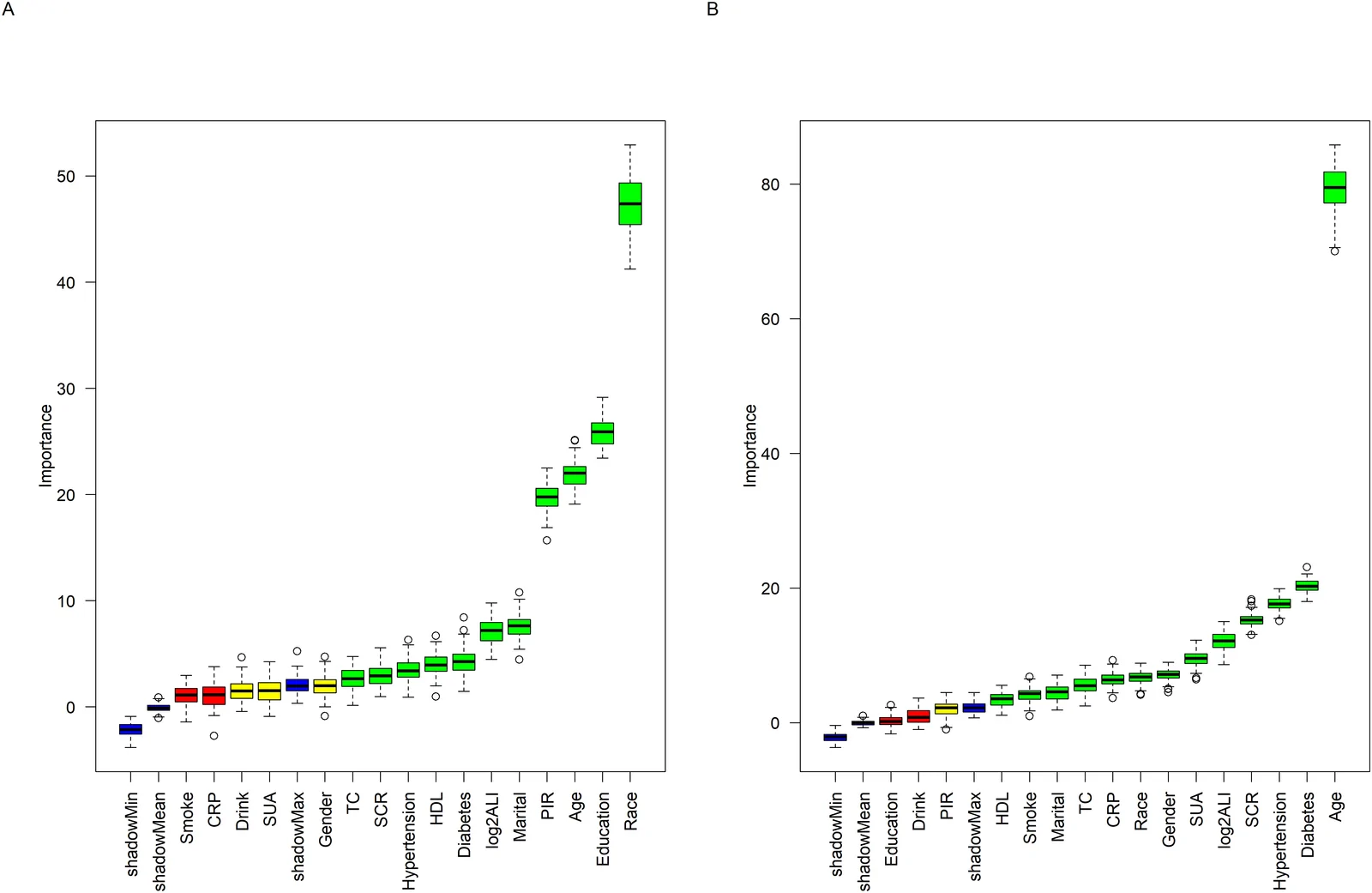
Feature selection based on Boruta algorithm: Boxplot of the importance distribution of each variable, arranged in descending order of the median importance, green indicates the significant variables confirmed by the algorithm. **(A)** The study of ALI and (H) pylori infection; **(B)** The study of ALI and mortality.

All analyses were performed using R software (version 4.3.3). To account for the complex sampling design and miss-response bias, sampling weights provided by the National Health and Nutrition Examination Survey were used. Sampling weights (WTMEC2YR for mortality analysis, and the appropriate interview/examination weights for the prevalence analysis), primary sampling units (SDMVPSU), and stratification variables (SDMVSTRA) were incorporated into all models. To minimize selection bias and handle varying degrees of missing data (**Supplementary Table S1**), the “mice” R package was used for imputation and iteration of covariates, with missing values handled through the predictive mean matching approach. Five imputed datasets were generated and analyzed separately, with final estimates pooled using Rubin’s rules. Since the proportion of missing data for all variables was within 15%, it was deemed unlikely to affect the results. Participants were stratified by *H. pylori*-positive and negative groups for baseline comparisons. Continuous variables are presented as median (interquartile range, IQR), while categorical variables are reported as frequencies and percentages. To clarify the relationships between ALI and *H. pylori* infection and mortality, we built multivariate logistic regression and Cox regression models with adjustment for confounding variables. Logistic regression results were reported as odds ratios (OR), and Cox regression results as hazard ratios (HR), both with 95% confidence intervals (95% CI). To identify the variables most associated with *H. pylori* infection and all-cause mortality, we use Boruta algorithm, a random forest-based feature selection method (**Figure 2**). Model 2 includes the first five relevant variables other than log2ALI, and model 3 includes all variables. In the study of ALI and *H. pylori* infection, model 1 was unadjusted for any covariates. Model 2 adjusted for age, race, education level, marital status, and economic status. Model 3 is further adjusted for diabetes, hypertension, HDL, SCR and TC based on Model 2. In the study of ALI and mortality, model 1 was unadjusted for any covariates. Model 2 adjusted for age, diabetes, hypertension, SCR and SUA. Model 3 further adjusted for gender, race, marital status, smoking status, CRP, TC and HDL. For prevalence analysis of the hospital sample, Model 1 was unadjusted; Model 2 adjusted for gender, age, education level, and marital status; Model 3 further adjusted for smoking status, hypertension, and diabetes, based on Model 2.

**Figure 3:**
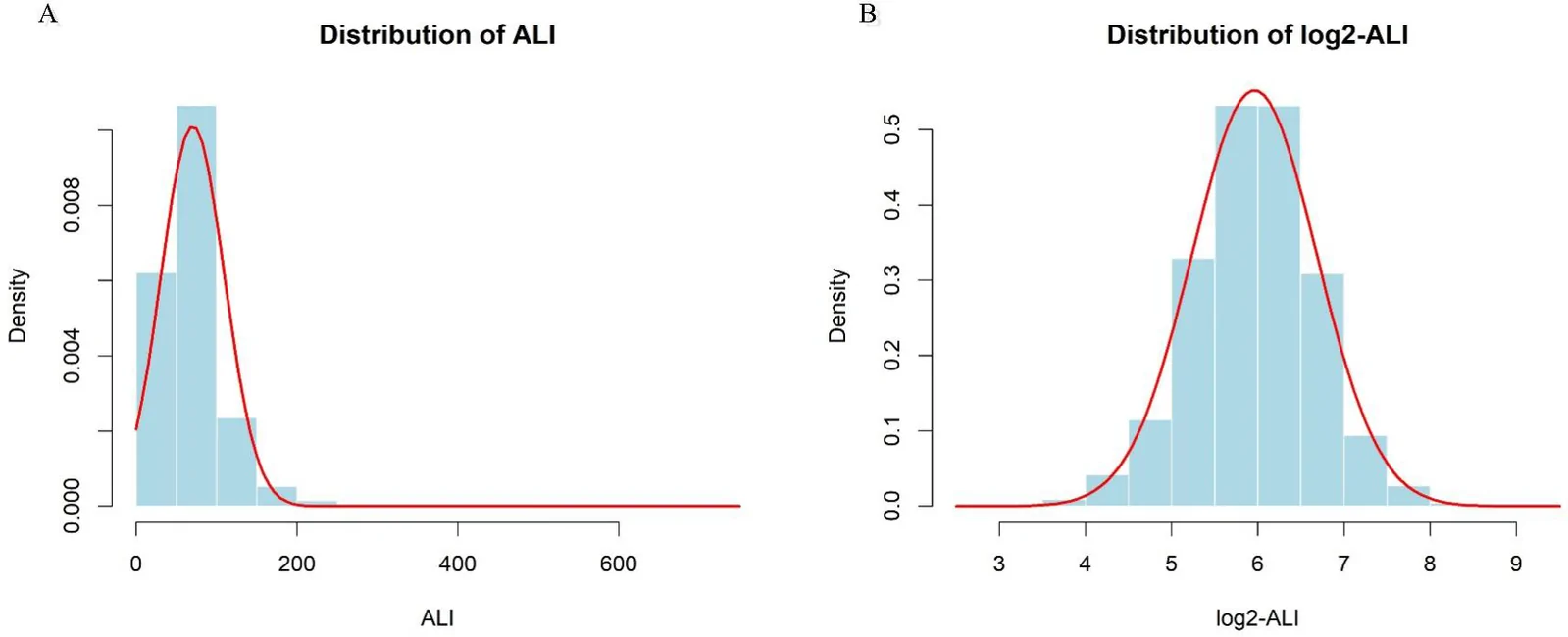
**(A)** Distribution of ALI, showing a right-skewed pattern. **(B)** Distribution of log2-ALI, following a normal distribution.

Multicollinearity was assessed by calculating the variance inflation factor (VIF) for all covariates in the models (**Supplementary Tables S2–S4**). All VIFs were <5, indicating no significant multicollinearity (American Diabetes Association Professional Practice Committee, 2024). Restricted cubic splines (RCS) were simultaneously used to evaluate potential nonlinear relationships. Kaplan–Meier survival curves were employed to describe the relationship between ALI and all-cause mortality in *H. pylori*-infected patients. Notably, during regression analysis, ALI was log2-transformed due to its right-skewed distribution (**Figure 3**).

Subgroup analyses were performed across predefined subgroups including gender, age, race, BMI, smoking status, and alcohol consumption to explore the consistency of the associations. As recommended in reporting guidelines for subgroup analyses, performing multiple independent statistical tests may elevate the risk of Type I error (Wang et al., 2007; Kim, 2019). In this study, Bonferroni correction was applied to reduce false-positive risk, comparing interaction *P* with a corrected significance threshold of α = 0.05/6 = 0.0083 (Bland and Altman, 1995).

Mediation analysis was used to test whether HbA1c is associated with ALI and *H. pylori* prevalence/mortality. For mediation analysis of HbA1c in *H. pylori* prevalence, HbA1c was the dependent variable and ALI the exposure variable in the mediator model, with all covariates from Model 3 included. In the outcome model, *H. pylori* infection was the dependent variable, with ALI and HbA1c as exposures and covariates identical to the mediator model. For mediation analysis of HbA1c in mortality, the mediator model was consistent with the above; in the outcome model, *H. pylori*-related mortality was the dependent variable, with all covariates from Model 3 included and other components unchanged. It should be noted that this study is a cross-sectional survey where exposure, mediator, and outcome were measured simultaneously, precluding clarification of causal sequence and making unmeasured confounding difficult to control. Thus, the “proportion mediated” reflects statistical overlap among variables rather than true mediation in a causal pathway.

## 3 Results

### 3.1 Baseline characteristics of participants

A total of 3,718 participants were enrolled in this study, among whom 1,688 were *H. pylori*-positive, giving an infection rate of 45.4%. Based on the weighted estimation, the sample corresponded to 170,899,745 U.S. adults. In the weighted sample, the prevalence was approximately 31.0 ± 1.2%. Finally, 1688 patients were included in the all-cause mortality cohort, corresponding to a total of 53,024,633 adults with *H. pylori* across the United States. Baseline characteristics are presented in **Table 1** below. The proportion of elderly individuals in the *H. pylori*-positive group was significantly higher than that in the *H. pylori*-negative group. Gender distribution was balanced between the two groups, and most participants were non-Hispanic White. The *H. pylori*-positive group exhibited a significantly lower education level, with 75.1% having a high school education or below (vs. 49.4% in the *H. pylori*-negative group), as well as poorer economic status. The proportion of participants who were married or living with a partner was also higher in the *H. pylori*-positive group. The *H. pylori*-positive group had higher prevalences of chronic condition in diabetes and hypertension. This group also showed slightly higher TC but lower HDL and ALB. CRP was higher, but another marker of systemic inflammation, the NLR was lower. During the entire follow-up period, the *H. pylori*-positive group had a higher mortality rate.

**Table 1:** Baseline characteristics of the study participants by *H. pylori*-negative and *H. pylori*-positive status.

Variables	Total	*H. pylori*-negative	*H. pylori*-positive	*P*
Count, n	3718	2030	1688	
Gender, n (%)				
Female	1867 (50.2)	1034 (50.9)	833 (49.3)	0.352
male	1851 (49.8)	996 (49.1)	855 (50.7)	
Age, years	50.00 (35.00, 66.00)	46.00 (32.00, 64.00)	54.00 (40.00, 67.00)	<0.001
Age, n (%)				
< 60 years	2328 (62.6)	1364 (67.2)	964 (57.1)	<0.001
≥60 years	1390 (37.4)	666 (32.8)	724 (42.9)	
Race, n (%)				
Mexican American	1004 (27.0)	334 (16.5)	670 (39.7)	<0.001
Non-Hispanic White	1688 (45.4)	1265 (62.3)	423 (25.1)	
Non-Hispanic Black	686 (18.5)	291 (14.3)	395 (23.4)	
Other races	340 (9.1)	140 (6.9)	200 (11.8)	
Education level, n (%)				
High school and below	2270 (61.1)	1002 (49.4)	1268 (75.1)	<0.001
College or above	1448 (38.9)	1028 (50.6)	420 (24.9)	
Economic condition, n (%)				
PIR < 1	754 (20.3)	300 (14.8)	454 (26.9)	<0.001
PIR ≥ 1	2964 (79.7)	1730 (85.2)	1234 (73.1)	
Marital status, n (%)				
Married/Living with a partner	2308 (62.1)	1227 (60.4)	1081 (64.0)	<0.001
Widowed/Divorced/Separated	821 (22.1)	425 (20.9)	396 (23.5)	
Never married	589 (15.8)	378 (18.6)	211 (12.5)	
BMI, n (%)				
< 25 (kg/m2)	1190 (32.0)	697 (34.3)	493 (29.2)	0.004
25–30 (kg/m2)	1323 (35.6)	695 (34.2)	628 (37.2)	
≥30 (kg/m2)	1205 (32.4)	638 (31.4)	567 (33.6)	
Smoking status, n (%)				
Never smoker	1933 (52.0)	1089 (53.6)	844 (50.0)	0.056
Current smoker	781 (21.0)	402 (19.8)	379 (22.5)	
Former smoker	1004 (27.0)	539 (26.6)	465 (27.5)	
Drinking status, n (%)	2507 (67.4)	1410 (69.5)	1097 (65.0)	0.004
Diabetes, n (%)	486 (13.1)	202 (10.0)	284 (16.8)	<0.001
Hypertension, n (%)	1565 (42.1)	771 (38.0)	794 (47.0)	<0.001
MORTSTAT, n (%)	1225 (32.9)	591 (29.1)	634 (37.6)	<0.001
Follow-up time, month	234.00 (188.00, 242.00)	235.00 (207.00, 242.00)	233.00 (170.75, 242.00)	0.033
BMI (kg/m^2^)	27.45 (24.13, 31.51)	27.17 (23.80, 31.50)	27.80 (24.47, 31.53)	0.008
ALB (g/l)	4.50 (4.30, 4.70)	4.50 (4.30, 4.70)	4.40 (4.20, 4.60)	<0.001
NLR	1.97 (1.48, 2.64)	2.08 (1.57, 2.75)	1.88 (1.38, 2.50)	<0.001
TC (mmol/l)	201.00(176.00, 229.00)	200.00(176.00, 227.00)	203.00(177.75, 231.00)	0.037
HDL (mmol/l)	48.00 (40.00, 58.00)	48.00 (40.00, 60.00)	47.00 (39.00, 57.00)	<0.001
SCR (mmol/l)	0.70 (0.60, 0.90)	0.70 (0.60, 0.90)	0.70 (0.60, 0.90)	0.185
SUA (mmol/l)	5.20 (4.30, 6.30)	5.20 (4.30, 6.30)	5.30 (4.40, 6.40)	0.202
CRP (mg/l)	0.24 (0.10, 0.54)	0.22 (0.09, 0.52)	0.27 (0.11, 0.58)	<0.001

### 3.2 Association between ALI and *H. pylori* infection

Multivariate logistic regression results showed that higher ALI was a relevant factor for *H. pylori* infection (**Table 2**). In the final multivariable-adjusted model (Model 3), ALI is associated with a higher rate of *H. pylori* infection (OR = 1.35, 95% CI:1.03-1.75, *P* = 0.037). Additionally, log_2_ALI was divided into four levels (Q1 as the lowest, Q4 as the highest), with Q1 as the reference. In the fully adjusted model, Q4 group was not statistically significant compared to Q1 group.

**Table 2:** Multivariate logistic regression of the log_2_ALI with *H. pylori* infection.

log2.ALI	Model 1		Model 2		Model 3	
	OR (95% CI)	*P*	OR (95% CI)	*P*	OR (95% CI)	*P*
Continuous	1.33 (1.15,1.53)	0.001	1.36 (1.13,1.63)	0.005	1.35 (1.03,1.75)	0.037
Quartile						
Q1	Ref		Ref		Ref	
Q2	0.97 (0.72,1.31)	0.819	1.05 (0.78,1.42)	0.689	1.05 (0.75,1.48)	0.704
Q3	1.58 (1.21,2.08)	0.003	1.67 (1.16,2.40)	0.014	1.67 (1.09,2.54)	0.028
Q4	1.43 (1.06,1.93)	0.024	1.48 (1.01,2.17)	0.048	1.47 (0.95,2.28)	0.07
*P* for trend		0.003		0.012		0.019

**Figure 4:**
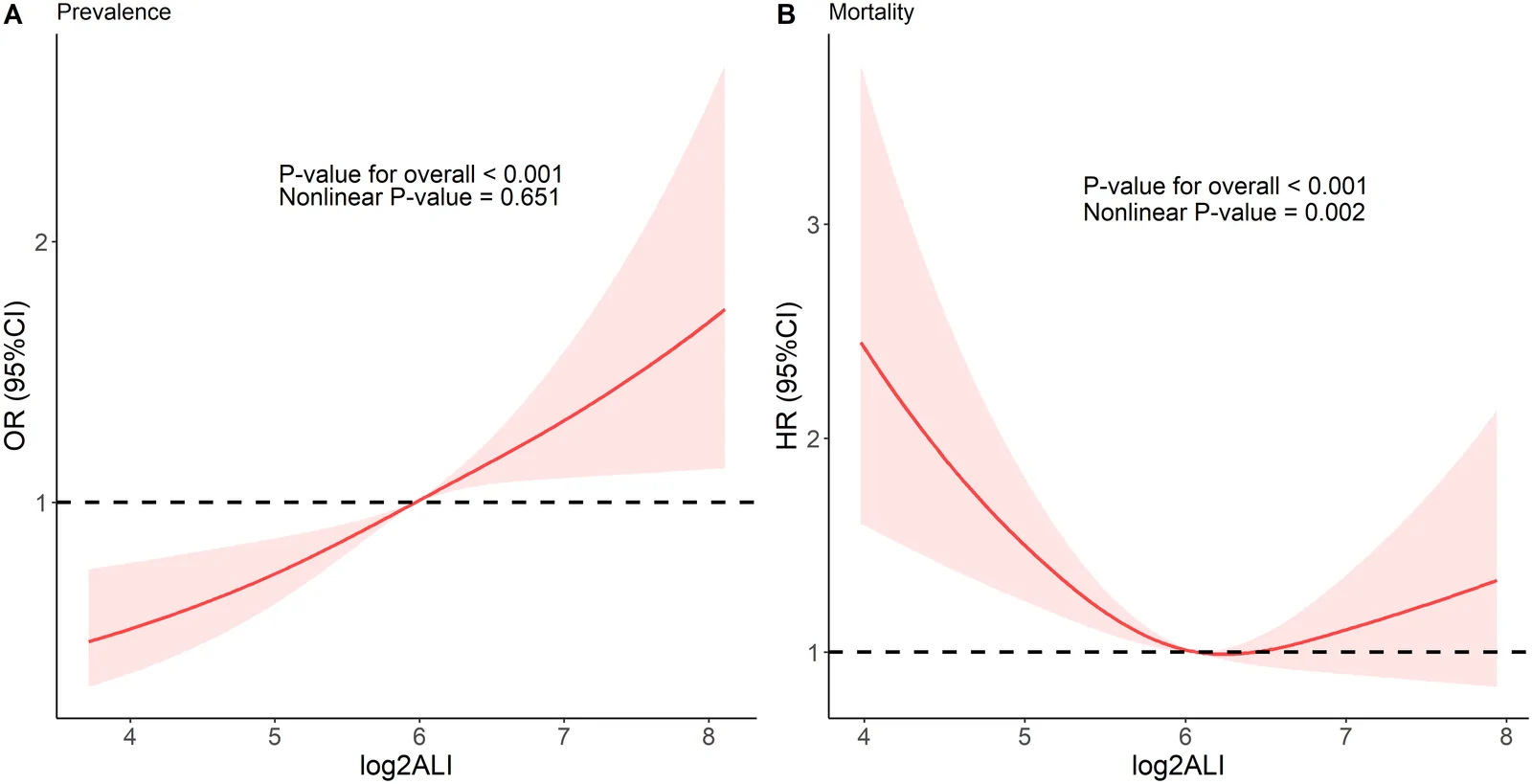
**(A)** The RCS curves between log_2_ALI and *H. pylori* infection risk of the NHANES database. **(B)** The RCS curves between log_2_ALI and all-cause mortality risk of the NHANES database. The red solid lines represent the predicted values and the red shaded areas represent 95% CI.

RCS curves were applied to assess the dose-response relationship between log_2_ALI and the risk of *H. pylori* infection. In **Figure 4A** (relationship between log_2_ALI and *H. pylori* infection risk), the *P* for the nonlinearity test was 0.651, indicating an approximately linear dose-response relationship between log_2_ALI and *H. pylori* infection risk. As log_2_ALI levels increased, the prevalence of *H. pylori* infection increased.

Model 1 was unadjusted for any covariates. Model 2 adjusted for age, race, education level, marital status, and economic status. Model 3 adjusted for age, race, education level, marital status, economic status, diabetes, hypertension, HDL, SCR and TC.

### 3.3 Association between ALI and all-cause mortality

**Figure 5:**
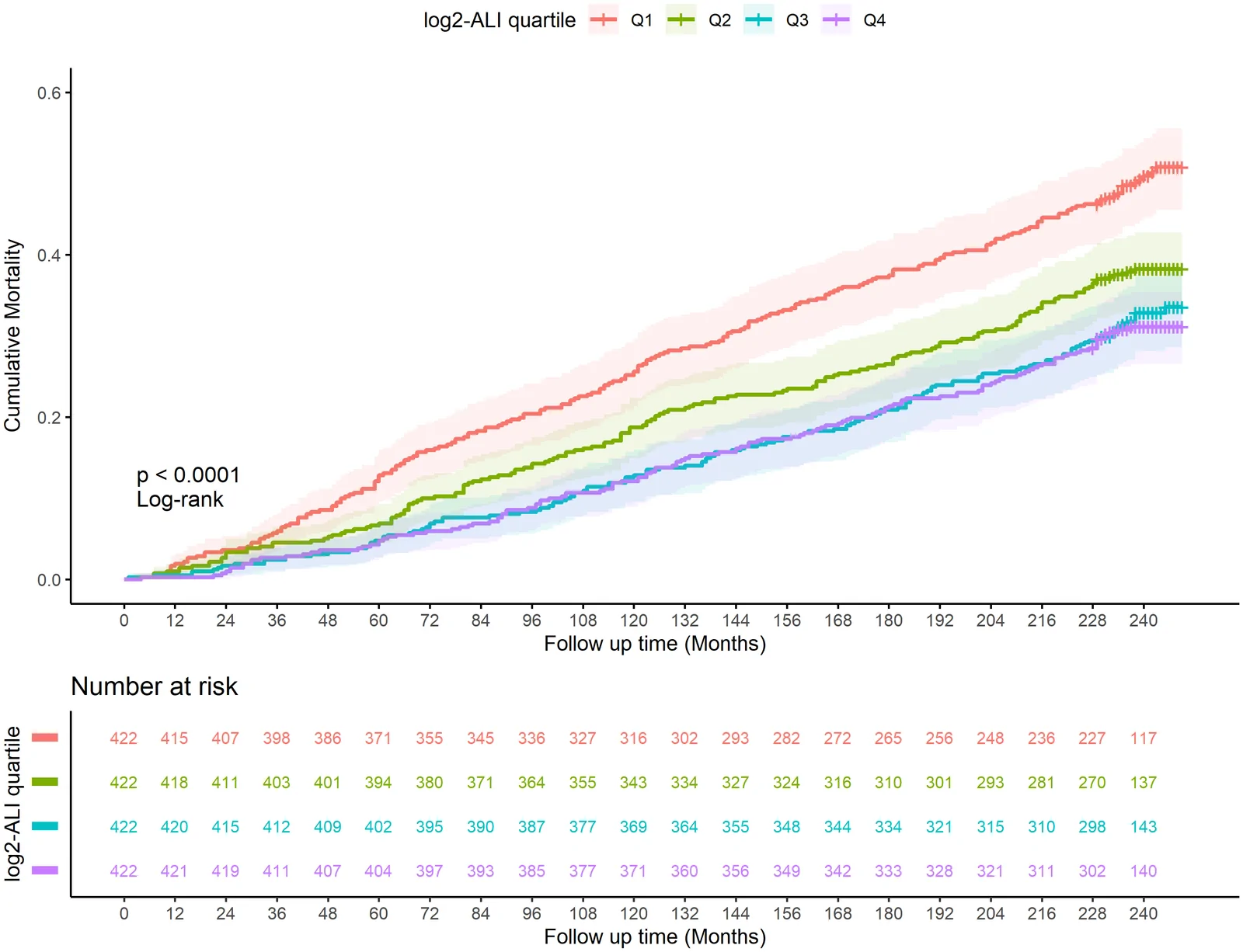
Kaplan-Meier cumulative mortality curves with *H. pylori* infection classified by different log_2_ALI levels.

Cox regression results showed that higher ALI levels were associated with longer survival time in *H. pylori*-infected patients (**Table 3**). In Model 3, higher log_2_ALI values were associated with lower all-cause mortality (HR = 0.83 95% CI: 0.70–0.98; *P* = 0.028). Survival analysis with Log_2_ALI grouped by quartiles demonstrated that all HRs of each quartile group (Q2-Q4) relative to Q1 were less than 1 (**Figure 5**). However, the magnitude of risk reduction did not show a monotonic decreasing trend as ALI grade increased, indicating that the association was not strictly dose-response. Additionally, the cumulative mortality curve for the low Log_2_ALI group remained at the top throughout, indicating the worst survival rate. In the early follow-up period, separation between different Log_2_ALI level groups was most pronounced, suggesting that the relevance was particularly prominent in the early stage.

**Table 3:** Cox regression of Log_2_ALI with all-cause mortality risk.

log2.ALI	Model 1		Model 2		Model 3	
	HR (95% CI)	*P*	HR (95% CI)	*P*	HR (95% CI)	*P*
Continuous	0.68 (0.54,0.85)	<0.001	0.78 (0.65,0.93)	0.006	0.83 (0.70,0.98)	0.028
Quartile						
Q1	Ref		Ref		Ref	
Q2	0.69 (0.47,1.00)	0.051	0.73 (0.55,0.98)	0.033	0.83 (0.60,1.13)	0.236
Q3	0.67 (0.51,0.87)	0.003	0.74 (0.61,0.88)	<0.001	0.84 (0.69,1.02)	0.073
Q4	0.56 (0.38,0.81)	0.002	0.73 (0.51,1.04)	0.082	0.80 (0.55,1.17)	0.249
*P* for trend		<0.001		0.055		0.212

Model 1 was unadjusted for any covariates. Model 2 adjusted for age, diabetes, hypertension, SCR and SUA. Model 3 adjusted for age, gender, race, marital status, smoking status, diabetes, hypertension, SCR, SUA, CRP, TC and HDL.

RCS curves revealed an approximate L-shaped nonlinear relationship between log2ALI and all-cause mortality risk. In the descending segment of the curve (low to moderate log_2_ALI levels), higher log_2_ALI was associated with reduced mortality risk; after crossing the inflection point (log_2_ALI =5.44), the curve began to rise slowly (**Figure 4B**). The first half of the association was significant, and the second half of the curve suggested that there might be a slight upward trend of risk in the high value area, but the threshold association analysis showed that this upward segment did not reach statistical significance (**Table 4**).

**Table 4:** Threshold association analysis of ALI and mortality risk in patients with Helicobacter pylori infection.

Threshold association analysis	Adjusted HR (95%CI)	*P*
Mortality risk		
Inflection point = 5.44		
Log_2_ALI ≤5.44	0.49 (0.37, 0.65)	<0.001
Log_2_ALI >5.44	1.01 (0.86, 1.18)	>0.9
Log-likelihood ratio test		<0.001

### 3.4 Subgroup analysis

**Figure 6:**
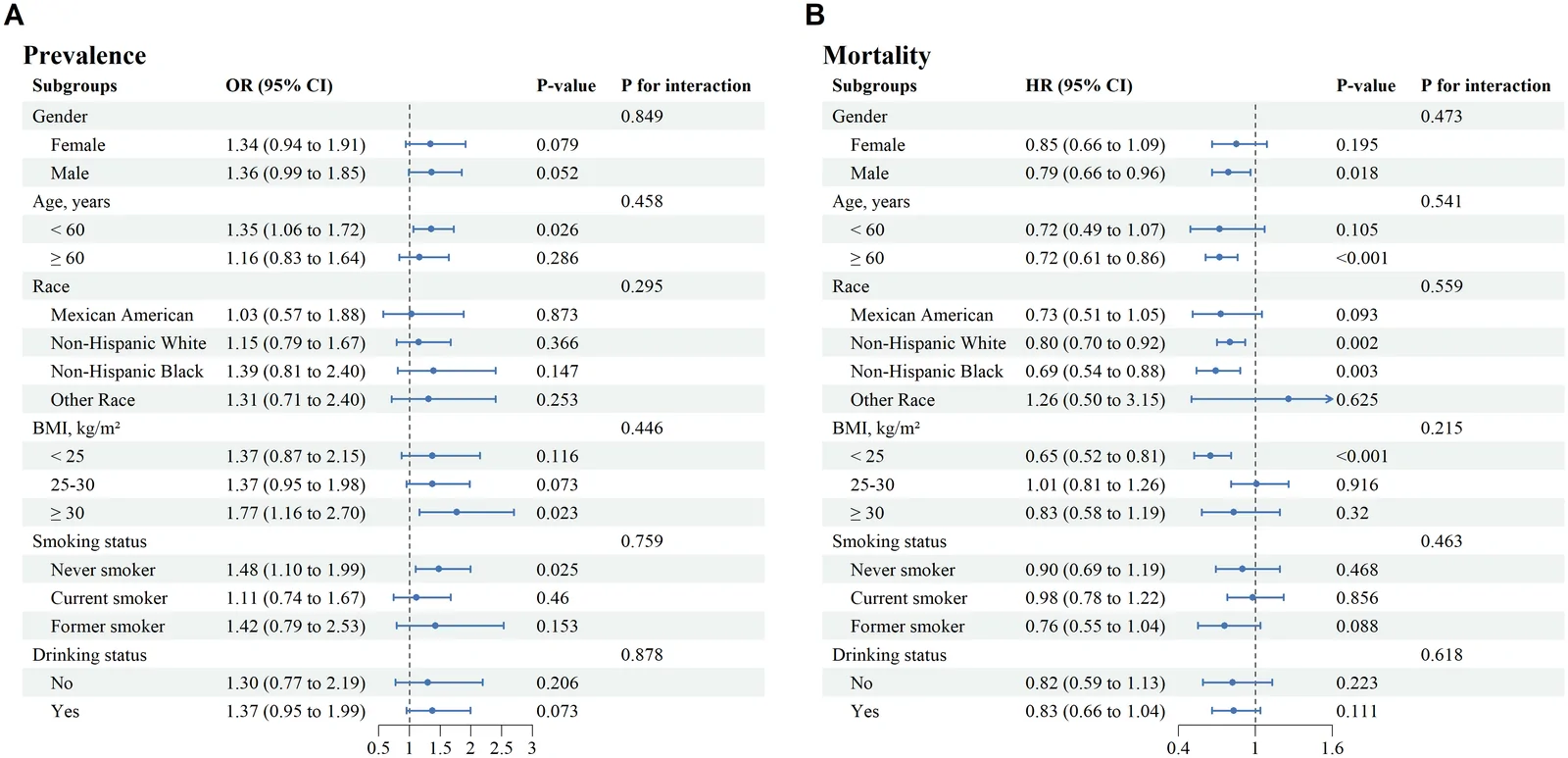
Each subgroup analysis was adjusted for gender, age, race, BMI, smoking status, and alcohol consumption status. The Bonferroni corrected α = 0.0083. **(A)** log_2_ALI with the risk of *H. pylori* infection. **(B)** log_2_ALI with the all-cause mortality.

In the subgroup analysis of ALI and the prevalence of *H. pylori* infection (**Figure 6A**), the interaction test showed that no significant interaction was found for all stratification factors (gender, age, race, BMI, smoking status, and drinking status) (all interaction *P* > 0.05). The association between ALI and *H. pylori* infection was consistent among the above subgroups. Within specific subgroups, age < 60 years (OR = 1.35, 95%CI: 1.06-1.72, *P* = 0.026), BMI ≥ 30 kg/m^2^ (OR = 1.77, 95%CI: 1.16-2.70, *P* = 0.023) and never smokers (OR = 1.48, 95%CI: 1.10-1.99, *P* = 0.025), ALI was positively associated with the prevalence of *H. pylori* infection. In the subgroup analysis of ALI and all-cause mortality (**Figure 6B**), no significant interactions were observed for all stratification factors (*P* > 0.05 for all interactions). With respect to the risk of death, the inverse association between ALI and all-cause mortality was significant in the following subgroups: Male (HR = 0.79,95%CI: 0.66-0.96, P = 0.018); Age ≥ 60 years old (HR = 0.72, 95%CI: 0.61-0.86, P < 0.001); BMI < 25kg/m^2^ (HR = 0.65, 95%CI: 0.52-0.81, P < 0.001).

### 3.5 Exploratory path coefficients

**Figure 7:**
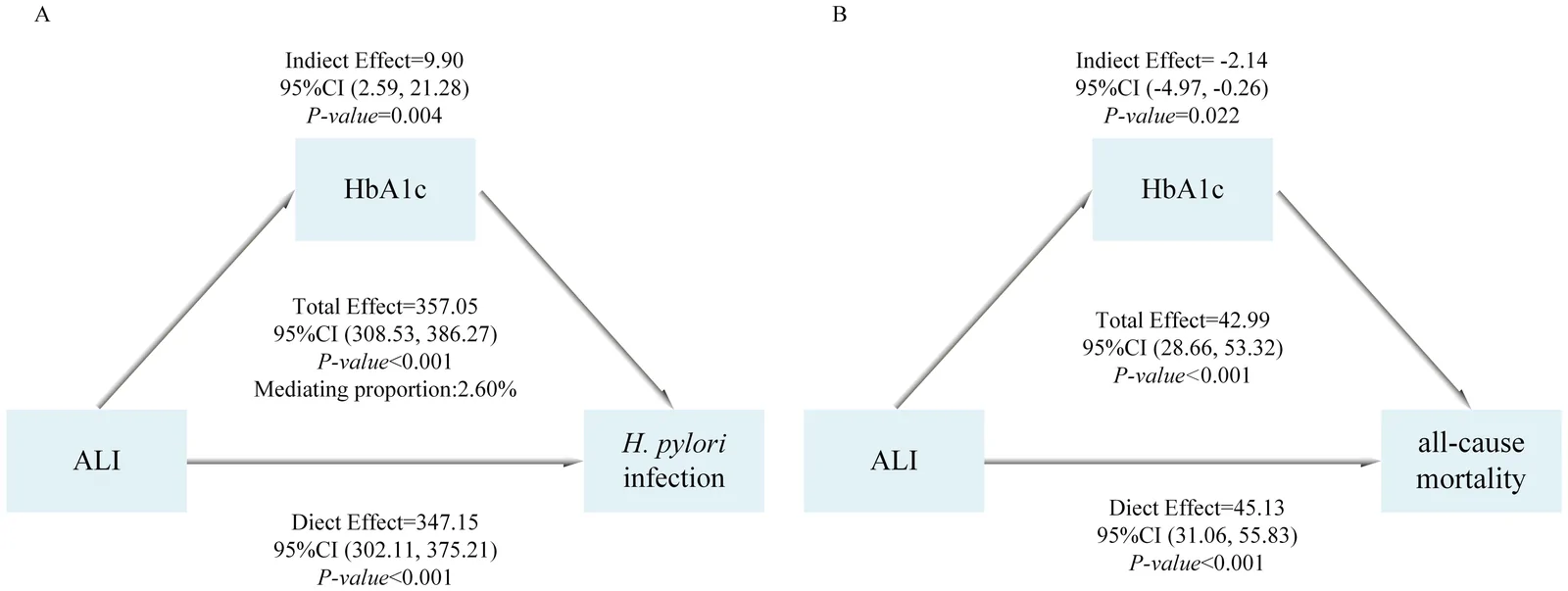
**(A)** The mediating effects of HbA1c on the relationship between ALI and *H. pylori* infection. The mediating analysis adjusted for age, race, education level, marital status, hypertension, diabetes, HDL-C, TC and SCR. **(B)** The mediating role of HbA1c in the association between ALI and all-cause mortality. The mediating analysis adjusted for gender, age, race, marital status, smoking status, hypertension, diabetes, HDL, TC, SCR, SUA and CRP.

To evaluate the mediating role of HbA1c in the association between ALI and *H. pylori* infection/prognosis, we calculated path coefficients using the bias-corrected Bootstrap method (1,000 resamplings). As illustrated in **Figure 7A**, the direct effect of ALI on *H. pylori* infection was 347.1, the indirect effect of ALI on *H. pylori* infection through HbA1c was 9.90. HbA1c mediated about 2.6% of the total effect of ALI on *H. pylori* infection (*P* = 0.004). This is an observed statistical overlap and not a causal mediator. In the all-cause mortality analysis, the direct effect of ALI on prognosis was 45.13 and the indirect effect of HbA1c on prognosis was -2.14. Mediation proportions were not routinely calculated because indirect effects were in the opposite direction to direct effects. (**Figure 7B**).

### 3.6 Independent duplicate cohort

For the independent duplicate cohort, a total of 575 patients were enrolled, of whom 194 (33.7%) were positive for *H. pylori.* Baseline characteristics of participants stratified by *H. pylori*-positive and *H. pylori*-negative status are presented in **Table 5**. Baseline analysis revealed that the proportion of men was higher in the *H. pylori*-positive group, and the median BMI was higher in the *H. pylori*-positive group than in the *H. pylori*-negative group. In terms of laboratory indicators, the ALB level was lower and LYC was higher in the positive group. The NLR was lower in the positive group than in the negative group, a trend consistent with the findings in the NHANES database. As the core indicators of this study, ALI and its log transformation value log2ALI in the *H. pylori*-positive group were significantly higher than those in the negative group. In addition, there were no significant differences in age, education level, marital status, smoking and drinking status, prevalence of diabetes and hypertension between the two groups, indicating that the above factors were well comparable between the two groups.

**Table 5:** Baseline characteristics of the study participants by *H. pylori*-negative and *H. pylori*-positive status in independent duplicate cohort.

Variables	Total	*H. pylori*-negative	*H. pylori*-positive	*P*
Count, n	575	381	194	
Gender, n (%)				0.012
Female	316 (55.0)	224 (58.8)	92 (47.4)	
male	259 (45.0)	157 (41.2)	102 (52.6)	
Age, years	58.00 (50.00, 67.00)	59.00 (49.00, 68.00)	58.00 (50.00, 66.00)	0.99
Education, n (%)				0.075
High school and below	345 (60.0)	239 (62.7)	106 (54.6)	
College or above	230 (40.0)	142 (37.3)	88 (45.4)	
Marital status, n (%)				0.28
Married/Living with a partner	530 (92.2)	348 (91.3)	182 (93.8)	
Never married	29 (5.0)	23 (6.0)	6 (3.1)	
Divorced/Separated/Widowed	16 (2.7)	10 (2.6)	6 (3.1)	
BMI, kg/m2	22.20 (20.20, 24.60)	22.00 (19.70, 24.10)	22.90 (20.83, 25.10)	0.001
Smoking status, n (%)				0.206
Smokers(present and past)	76 (13.2)	45 (11.8)	31 (16.0)	
Never smokers	499 (86.8)	336 (88.2)	163 (84.0)	
Drinking status, n (%)				0.987
No	544 (94.6)	361 (94.8)	183 (94.3)	
Yes	31 (5.4)	20 (5.2)	11 (5.7)	
Diabetes, n (%)	70 (12.2)	40 (10.5)	30 (15.5)	0.113
Hypertension, n (%)	144 (25.0)	88 (23.1)	56 (28.9)	0.159
ALB, g/l	43.70 (40.72, 46.16)	44.10 (41.02, 46.20)	42.90 (39.54, 46.00)	0.016
NE, 10^9^/l	3.67 (2.94, 4.66)	3.78 (3.01, 4.90)	3.51 (2.81, 4.34)	0.007
LYC, 10^9^/l	1.60 (1.30, 1.90)	1.56 (1.26, 1.81)	1.69 (1.37, 2.12)	<0.001
NLR	2.35 (1.84, 2.97)	2.45 (1.93, 3.15)	2.13 (1.70, 2.73)	<0.001
ALI	40.85 (31.82, 54.95)	39.81 (29.23, 52.51)	43.84 (34.17, 59.58)	<0.001
log2ALI	5.35 (4.99, 5.78)	5.32 (4.87, 5.71)	5.45 (5.09, 5.90)	<0.001

**Figure 8:**
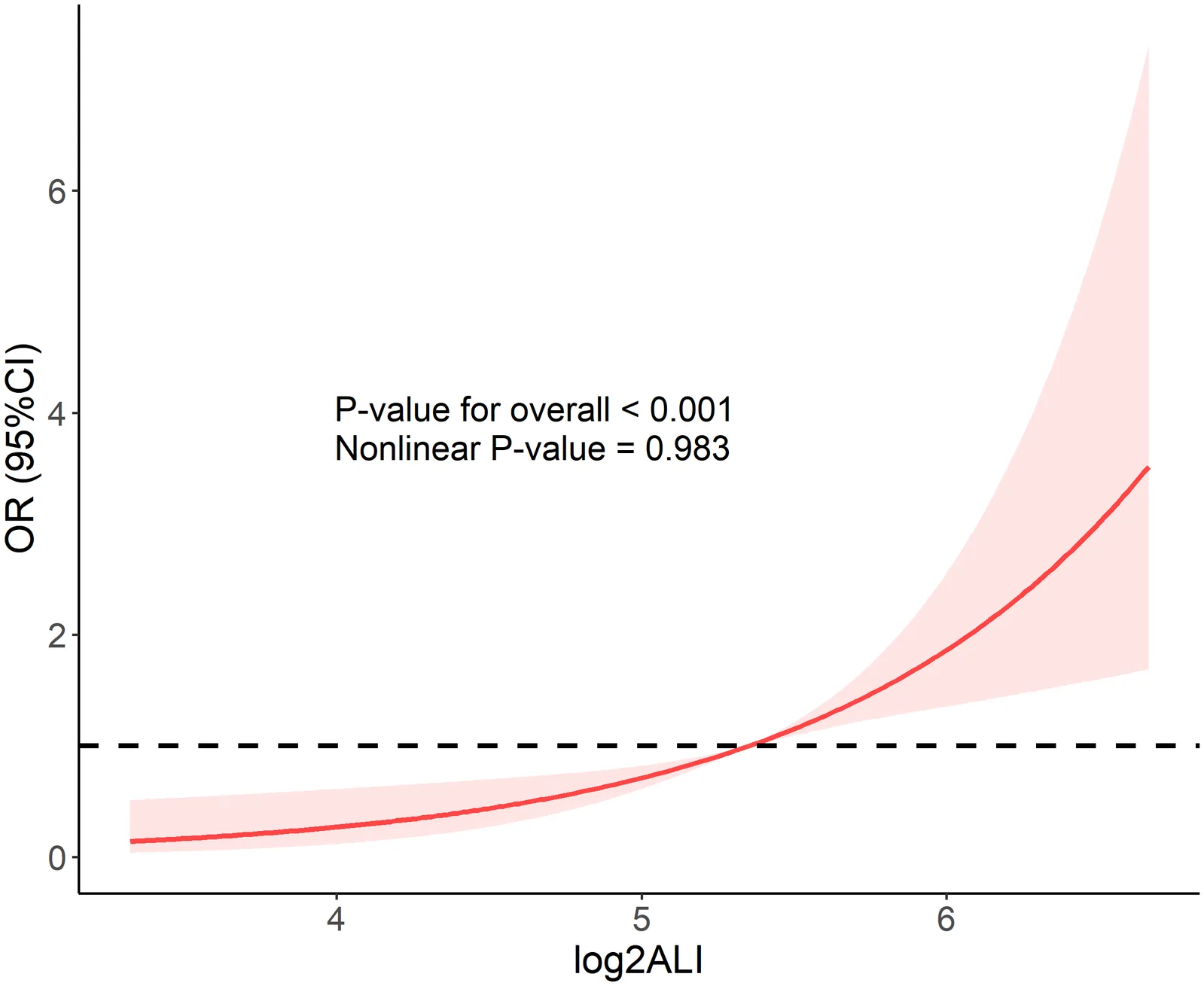
The RCS curves between log_2_ALI and *H. pylori* infection risk of the independent duplicate cohort. The red solid lines represent the predicted values and the red shaded areas represent 95% CI.

Independent duplicate cohort using our hospital data yielded consistent results. In the final multivariable-adjusted model, the probability of *H. pylori* infection was found to increase with the increase of log2ALI (OR = 1.94, 95% CI: 1.55–2.45, *P* < 0.001) (**Table 6**). When Log_2_ALI was grouped by quartiles (Q1–Q4) for analysis, in the final model (Model 3), the infection rate of *H. pylori* showed a significant upward trend with increasing Log_2_ALI quartiles (*P* for trend<0.001). Compared with Q1, group Q2 and group Q4 were significantly higher. Although the OR value of Q3 group decreased slightly compared with Q2 group and did not reach statistical significance, the overall linear trend test (*P* < 0.001) was still significant. The RCS curve of the independent duplicate cohort indicates that the *P* of the nonlinear test is 0.983, which also suggests that there is an approximately strict linear dose-response relationship between log_2_ALI and the likelihood of *H. pylori* infection. As the level of log_2_ALI increases, the likelihood of *H. pylori* infection (measured by OR value) showed a monotonous increasing trend (**Figure 8**).

**Table 6:** Multivariate logistic regression of the log_2_ALI with *H. pylori* infection in independent duplicate cohort.

log2.ALI	Model 1		Model 2		Model 3	
	OR (95% CI)	*P*	OR (95% CI)	*P*	OR (95% CI)	*P*
Continuous	1.93 (1.56,2.43)	<0.001	1.91 (1.54,2.42)	<0.001	1.94 (1.55,2.45)	<0.001
Quartile						
Q1	Ref		Ref		Ref	
Q2	2.02 (1.21,3.40)	0.007	2.07 (1.23,3.51)	0.006	2.05 (1.22,3.48)	0.007
Q3	1.31 (0.77,2.24)	0.326	1.32 (0.77,2.28)	0.311	1.32 (0.77,2.28)	0.31
Q4	2.93 (1.77,4.91)	<0.001	2.87 (1.72,4.85)	<0.001	2.93 (1.76,4.97)	<0.001
*P* for trend		<0.001		<0.001		<0.001

## 4 Discussion

Through systematic analysis of the NHANES database and incorporation of a cross-sectional sample of the Chinese population as a consistency check, this study is the first to comprehensively reveal the role of the ALI in *H. pylori*-infected individuals and its complex association with prognosis. Our analyses found that higher levels of ALI were associated with a higher rate of *H. pylori* infection. Among *H. pylori* infected individuals, higher ALI levels were associated with a lower risk of all-cause death, and there was a threshold for this protective association: When ALI was at a medium or low level, the mortality risk decreased significantly with the increase of ALI. However, when log_2_ALI exceeded 5.44, the mortality risk tended to plateau or even showed a slight upward trend, although the upward trend did not reach statistical significance. The positive association of *H. pylori* infection was consistently replicated in the independent duplicate cohort with a strict linear dose-response relationship. Mediation analysis suggested that HbA1c weakly mediated the association between ALI and *H. pylori* infection but had an opposite association on the death pathway. There was no significant interaction between subgroups, suggesting that the above association of ALI was generally stable across different demographic and lifestyle characteristics.

This study found that ALI was positively associated with *H. pylori* infection rate, which was also confirmed in the independent duplicate cohort, and showed a linear trend. Although the value of the ALI in the occurrence and prognosis of cancers such as lung cancer and gastric cancer has been fully recognized (Song et al., 2022; Catalano et al., 2024; Gao et al., 2024), its role in the context of *H. pylori* infection remains unclear. Most prior investigations have focused on the link between *H. pylori* and inflammatory states (Cai et al., 2021; Nagata et al., 2021; Fei et al., 2025), with some theoretical basis for the relationship between *H. pylori* and nutrition (Franceschi et al., 2014a; Aimasso et al., 2019). ALI is composed of body mass index, serum albumin and NLR, which is usually regarded as a comprehensive indicator of nutrition-inflammation (Zhong et al., 2025). In general, higher ALI reflects better nutritional reserve and lower systemic inflammation levels, which should be accompanied by lower susceptibility to infection (Al-Sawaf et al., 2023; GBD 2019 Chronic Respiratory Diseases Collaborators, 2023), but the opposite direction was observed in this study. This can be carefully understood from the following levels. First, *H. pylori* infection can change the nutritional status of the host by affecting the function of gastric mucosa and appetite and may also stimulate bone marrow hematopoiesis through persistent low-grade inflammation (Malfertheiner et al., 2012; Yu et al., 2024), leading to changes in peripheral blood lymphocyte or neutrophil count. Both this study and the independent duplicate cohort showed that NLR in the *H. pylori* positive group was significantly lower than that in the *H. pylori* negative group, which was mainly caused by the relative or absolute increase in lymphocyte count. Therefore, it is the characteristics of immune response caused by *H. pylori* infection, especially the lymphocyte-dominated chronic inflammatory response, which lowers NLR and then pushes up ALI value in the equation. In other words, a higher ALI may be partly a consequence of *H. pylori* infection rather than a cause. Secondly, a large number of epidemiological evidence has shown that *H. pylori* infection is positively associated with obesity and metabolic syndrome, and its mechanism may involve the regulation of appetite hormones such as ghrelin and leptin (He et al., 2025; Jiang et al., 2025; Ye et al., 2025). In this study, serum albumin was slightly lower in the *H. pylori* positive group, the independent duplicate cohort showed that the median body mass index was higher in the *H. pylori* positive group. Thus, higher ALI values may simply reflect a metabolic state of overnutrition, which may often occur simultaneously with *H. pylori* infection. Therefore, the positive association between ALI and *H. pylori* infection is more likely to be a cross-sectional reflection of the host immune-metabolic state, and it is not suitable to directly interpret that the increase of ALI will promote infection.

In contrast to the infection association, higher ALI level was associated with reduced risk of all-cause mortality in individuals infected with *H. pylori*, indicating that this indicator can still play a role in poor prognosis stratification in the infected population. No significant interactions were found for all stratification factors in the subgroup analysis, indicating that the association between ALI and *H. pylori* infection was consistent across the subgroups we studied. ALI was originally constructed as a prognostic index for cancer patients, which can comprehensively reflect the degree of nutrient depletion and inflammatory load of the host (Qiu et al., 2024; Zhu et al., 2026b). Low ALI implies low body mass index, low albumin, and high NLR, pointing, respectively, to malnutrition, protein-energy wasting, and neutrophil-dominated systemic inflammation (Zhang et al., 2023; Ma et al., 2025), all of which are recognized risk factors for poor outcomes. In this study, the cumulative mortality curve in the low ALI group was always at the highest position, especially in the early follow-up period, which showed obvious separation, suggesting that malnutrition-inflammation state marked by low ALI may show a positive correlation with earlier death events in *H. pylori* infected patients. With the increase of log_2_ALI near 5.44, the mortality risk did not continue to decrease, and the curve even showed a slightly upward nonlinear turn. This L-shaped relationship deserves attention. Presumably, in the very high ALI, obesity-related metabolic burden such as insulin resistance and cardiovascular risk (Smith et al., 2019), due to high BMI and other inflammatory patterns that may be masked gradually offset the positive advantage of good nutrition and low NLR (Buonacera et al., 2022). It is worth noting that the subsequent threshold analysis showed that the upward trend of the high value segment was not statistically significant, so the existing evidence only supports that too low ALI is a clear risk signal, and the additional risk of too high ALI needs to be verified in a larger sample and longer follow-up. In addition, it is still an observational association and cannot be directly equivalent to the causal association of the intervention target.

The data based on Chinese hospitals not only reproduced the positive association between ALI and *H. pylori* infection, but also had the same association direction, and even stronger association strength, showing a strict linear trend. This somewhat reduces the chance that the results are due to a single population, a particular test, or a particular time period. There were significant differences in *H. pylori* detection methods, population composition, overall infection rate, lifestyle background and the period between the two cohorts. Consequently, we interpret this as a consistency check rather than an external validation. However, the association between increased ALI and increased risk of infection remained stable, and NLR was significantly lower in the *H. pylori* positive group. This reinforces the universality of the explanation that *H. pylori* infection may alter ALI by affecting the distribution of lymphocytes and neutrophils. The lack of follow-up data on death in the independent duplicate cohort is a limitation. Therefore, the prognostic association between ALI and infection needs to be prospectively validated in more non-US populations.

Mediation analyses assessing HbA1c were exploratory. Because ALI, HbA1c, and *H. pylori* status were measured simultaneously, the observed statistical overlap does not constitute evidence of causal mediation. Mediation analysis revealed that HbA1c had a positive indirect effect between ALI and *H. pylori* infection of about 2.6%, which was statistically significant, indicating that the role of glucose metabolism pathway was very limited. Almost all the major associations between ALI and infection came from the direct pathway, further suggesting that the information contained in ALI goes far beyond the scope of glucose metabolism and may cover a wider network of immune-nutrition-metabolism. However, in the path analysis of all-cause mortality, the indirect effect was opposite to the direct effect, showing inconsistent mediation phenomenon, which may be due to the confounding effect or inhibitory effect of HbA1c on mortality outcome, suggesting that blood glucose control should not be simply regarded as a causal bridge for ALI to reduce the risk of death. Some of these results should be interpreted extremely conservatively.

The core advantages of our study stem from the use of the NHANES database for data analyses, paired with the inclusion of our hospital’s clinical data as consistency test, adjustment for multiple confounders, application of scientific statistical methods to explore, and comprehensive subgroup analysis to derive conclusions. However, several limitations should be noted: As a retrospective study, residual confounding cannot be entirely excluded despite adjustment for multiple factors. More importantly, observational studies can only reveal statistical associations between variables, not establish causality. Second, two cohorts differed in diagnostic methodology (IgG serology reflecting cumulative exposure in NHANES versus urea breath test reflecting active infection in the hospital cohort), temporal setting (1999–2000 versus 2025), and population structure (nationally representative U.S. sample versus single-center Chinese sample). These differences preclude direct quantitative comparison and mean that the Chinese cohort should be interpreted as an assessment of directional consistency rather than a strict external validation. Future studies require external validation in larger, multi-regional, multi-ethnic independent prospective sample to ensure broader applicability. Finally, as a composite index, the relative contributions of ALI components to the observed relationships remain unclear, necessitating more refined mechanistic studies in the future.

## 5 Conclusion

This study analyzed the relationship between inflammation index (ALI) and Helicobacter pylori (*H. pylori*) infection and all-cause mortality in patients with *H. pylori*. The results showed that higher ALI level was positively and robustly associated with an increased risk of *H. pylori* infection, and the consistency and linear trend of this association were further confirmed in the independent duplicate cohort. In patients with *H. pylori* infection, higher ALI level is associated with a reduced risk of all-cause mortality, and this association is particularly significant when ALI is low, suggesting that low ALI is independently associated with an increased long-term risk of death in patients with *H. pylori* infection. However, the current evidence only supports ALI as a potential epidemiological and prognostic marker but does not establish it as a reliable clinical biomarker for risk stratification. In the future, prospective cohort studies are urgently needed to dynamically monitor the evolution of ALI and its components at the beginning of infection diagnosis and incorporate the changes after eradication therapy and long-term mortality follow-up to clarify the practical value of ALI in the precise hierarchical management of *H. pylori* infection.

## Data Availability

The original contributions presented in the study are included in the article/**Supplementary Material**. Further inquiries can be directed to the corresponding author.
