# Supplementary material for: The association of the advanced lung cancer inflammation index with the risk of Helicobacter pylori infection and mortality: a study of populations from two countries
Source: Front Cell Infect Microbiol. 2026 Sep 10;16:1881537. doi: 10.3389/fcimb.2026.1881537 (PMC13601264; doi:10.3389/fcimb.2026.1881537)
Supplement: Supplementary file 1 [file Table1.docx]

Table S1: Distribution of variables with missing data.--NHANES

| Variable | Missing (n) | Percent (%) |
| --- | --- | --- |
| Education | 10 | 0.27 |
| PIR | 501 | 13.47 |
| Marital | 384 | 10.33 |
| Waist | 62 | 1.67 |
| SBP | 98 | 2.64 |
| DBP | 129 | 3.47 |
| Mean_sbp | 90 | 2.42 |
| Mean_dbp | 90 | 2.42 |
| TC | 7 | 0.19 |
| HDL | 8 | 0.22 |
| HbA1c | 1 | 0.03 |
| Smoke | 7 | 0.19 |

TableS2: Collinearity Statistics.(OR)--NHANES

| Variable | VIF |
| --- | --- |
| log2ALI | 1.08 |
| Age | 1.56 |
| Race | 1.07 |
| Education | 1.17 |
| Marital | 1.15 |
| PIR | 1.21 |
| Diabetes | 1.12 |
| Hypertension | 1.34 |
| HDL | 1.08 |
| TC | 1.09 |
| SCR | 1.05 |

TableS3: Collinearity Statistics.(HR)--NHANES

| Variable | VIF |
| --- | --- |
| log2ALI | 1.11 |
| Gender | 1.47 |
| Age | 1.16 |
| Race | 1.08 |
| Marital | 1.18 |
| TC | 1.08 |
| HDL | 1.2 |
| SCR | 1.05 |
| SUA | 1.19 |
| CRP | 1.07 |
| Smoke | 1.1 |
| Diabetes | 1.08 |
| Hypertension | 1.13 |

TableS4: Collinearity Statistics.(OR)—Hospital data

| Variable | VIF |
| --- | --- |
| log2ALI | 1.02 |
| Gender | 1.21 |
| Age | 1.3 |
| Education | 1.13 |
| Marital | 1.04 |
| Alcohol | 1.21 |
| Smoking | 1.32 |
| Diabetes | 1.2 |
| Hypertension | 1.31 |
